# A Mobile Phone–Based Intervention to Improve Mental Health Among Homeless Young Adults: Pilot Feasibility Trial

**DOI:** 10.2196/12347

**Published:** 2019-07-02

**Authors:** Stephen M Schueller, Angela C Glover, Anne K Rufa, Claire L Dowdle, Gregory D Gross, Niranjan S Karnik, Alyson K Zalta

**Affiliations:** 1 Department of Psychological Science University of California Irvine Irvine, CA United States; 2 Center for Behavioral Intervention Technologies Department of Preventive Medicine Northwestern University Chicago, IL United States; 3 Department of Psychiatry and Behavioral Sciences Rush University Medical Center Chicago, IL United States; 4 Stepwell Mental Health and Wellness Boulder, CO United States; 5 The Night Ministry Chicago, IL United States

**Keywords:** mental health, homelessness, telemedicine, treatment

## Abstract

**Background:**

Youth homelessness is a substantial issue, and many youths experiencing homelessness have mental health issues as both a cause and consequence of homelessness. These youths face many barriers to receiving traditional mental health services, and as a result, only a few youths experiencing homelessness receive any form of mental health care.

**Objective:**

This project aimed to develop and determine the feasibility and acceptability of engaging young adults (ie, individuals aged 18-24 years) experiencing homelessness in a remotely delivered mental health intervention. This intervention provided brief emotional support and coping skills, drawing from cognitive behavioral principles as an introduction into psychosocial support. The intervention was piloted in a homeless shelter network.

**Methods:**

A total of 35 young adults experiencing homelessness participated in a single-arm feasibility pilot trial. Participants received a mobile phone, a service and data plan, and 1 month of support from a coach consisting of up to 3 brief phone sessions, text messaging, and mobile mental health apps. We evaluated feasibility by looking at completion of sessions as well as the overall program and acceptability with satisfaction ratings. We also collected clinical symptoms at baseline and the end of the 1-month support period. We used validity items to identify participants who might be responding inappropriately and thus only report satisfaction ratings and clinical outcomes from valid responses.

**Results:**

Most participants (20/35, 57%) completed all 3 of their phone sessions, with an average of 2.09 sessions (SD 1.22) completed by each participant. Participants sent an average of 15.06 text messages (SD 12.62) and received an average of 19.34 messages (SD 12.70). We found higher rates of satisfaction among the participants with valid responses, with 100% (23/23) of such participants indicating that they would recommend participation to someone else and 52% (12/23) reporting that they were *very* or *extremely* satisfied with their participation. We found very little change from pre- to posttreatment on measures of depression (*d*=0.27), post-traumatic stress disorder (*d*=0.17), and emotion regulation (*d*=0.10).

**Conclusions:**

This study demonstrated that it was feasible to engage homeless young adults in mental health services in this technology-based intervention with high rates of satisfaction. We did not find changes in clinical outcomes; however, we had a small sample size and a brief intervention. Technology might be an important avenue to reach young adults experiencing homelessness, but additional work could explore proper interventions to deliver with such a platform.

**Trial Registration:**

ClinicalTrials.gov NCT03620682; https://clinicaltrials.gov/ct2/show/NCT03620682

## Introduction

Youth homelessness is a substantial issue. In 2018, 36,361 youths, that is, those younger than 25 years, experienced homelessness on a single night [1], and this number is higher when individuals experiencing housing instability or insufficient housing are included. Youth homelessness is a complex phenomenon that spans a continuum of experiences from street-based youth to shelters to unstable accommodations. In a previous literature review, we found that the stresses of homelessness have adverse consequences on physical and mental health among youth [2]. Individuals experiencing homelessness have high rates of mental illness and substance misuse, nearly twice as high as those among their housed peers [3]. Youth experiencing homelessness have disproportionate rates of emotional and behavioral problems [4-6]. Estimates suggest that nearly two-thirds of homeless youth meet criteria for a psychiatric disorder [7], with particularly high rates of depression and anxiety [8,9]. Unfortunately, the vast majority of homeless individuals have little or no access to comprehensive health care and virtually no access to mental health services. Many of the realities of homelessness make receiving mental health care, especially traditional face-to-face services, more challenging. For example, it is often difficult for individuals experiencing homelessness to make and keep scheduled appointment times given the frequency of other time-urgent demands that supersede scheduled activities.

As such, traditional mental health services may be ineffective at reaching and addressing the needs of this population. One complement to traditional mental health services that could help overcome some of these barriers would be technology-based treatment options [10]. Such options include resources such as teletherapy, text messaging, and mobile apps. Technology may be an especially promising strategy to engage hard-to-reach populations. For example, Fortney and colleagues [11] showed that a telemedicine outreach program for rural veterans increased engagement in post-traumatic stress disorder (PTSD) treatment. Moreover, technology may be especially suited for engaging youth given that they have mobile phones, and many report being online *almost constantly* [12]. Indeed, access to health care services is insufficient and much lower than the general population, but individuals experiencing homelessness, including youth and adults, have a level of access to mobile technologies comparable with similar aged peers. One-fourth of homeless young adults report using the internet for more than an hour a day (most often accessed via mobile devices) [13,14], and rates of mobile phone ownership are also high (eg, ranging from 44% to 62%) [15,16], with those aged between 18 to 29 years accounting for the top end of that range [17]. Mobile technology may be even more important for people who lack a fixed place of residence. Mobile phones allow for social connections, searching for resources, and entertainment (to pass time) that are important parts of survival when faced with homelessness or unstable housing.

Thus, technology-based resources have the potential to be useful for homeless youth. A first question is what types of resources might yield themselves to technology-based interventions in this population. A few studies have suggested that cognitive behavioral therapy (CBT) delivered in shelters can lead to significant decreases in depression and other mental health problems and improvements in self-efficacy [18,19]. However, these studies have demonstrated considerable barriers to engagement. In 1 study, only treatment completers displayed significant benefits, and over half of the young adults who began treatment discontinued after the first session [19]. Thus, evidence-based practices must be provided in ways that are appropriate and acceptable for the setting and population.

Notably, CBT principles are amenable to delivery both in very brief formats [20] and also via technology [21]. For example, Schleider and Weisz [22] showed that in a group of high-risk adolescents, a single-session computer-guided growth mindset intervention led to greater improvements in depression and anxiety than a supportive-therapy control with lasting effects. To our knowledge, there are no published studies providing mental health interventions to homeless youth via technology. Our previous work exploring the interest of homeless youth in the use of mobile technology for mental health purposes indicated that youth would be most eager to receive help in the form of emotional support, help with life decisions, managing day-to-day stressors, problem solving, advice, and dealing with difficulties related to homelessness [23]. Thus, we were interested in building a very brief intervention, leveraging CBT principles that could serve as an initial step to engage youth in mental health services and alleviate distress with support and coping skills. Given these goals, we deemed this intervention the Stepping Stone project.

In this study, we evaluated the feasibility, acceptability, and preliminary benefits of a multicomponent mobile phone–based intervention for homeless young adults. Although youth refers to those younger than 25 years, we only engaged those in the age group of 18 to 24 years who were receiving services from a homeless shelter network focused on this age group. Feasibility was determined based on the young adults’ engagement with various components of the program. Acceptability was determined based on satisfaction ratings of the program as a whole as well as its different components. Finally, preliminary benefits were explored by examining changes in symptoms of depression, PTSD, and emotion regulation. To do so, we conducted a single-arm pilot trial of our multicomponent intervention recruiting participants from an urban homeless shelter network.

## Methods

### Participants

Participants were recruited from January 2016 to November 2017 from a homeless shelter network located in Chicago, IL, United States. Young adults responded to flyers distributed throughout the shelters or were referred to the study by their case manager. Potential participants were screened at the shelter by a member of the study staff. If eligible and interested, the participant went through the informed consent process and then filled out a series of baseline assessments on an iPad. These assessments collected information about demographics, trauma history, experience with technology and with the mental health care system, and current psychological symptoms. After completion of the surveys, they were provided with a mobile phone with an activated data plan, phone case, and headphones. Participants were then shown how to use the 3 study apps and were given tips on conserving data usage and using the phone responsibly and safely in an urban space including how best to secure their phones both physically (eg, keeping it safely concealed in certain spaces) and digitally (eg, setting up a password).

The eligibility criteria included (1) aged between 18 and 24 years, (2) English-speaking, and (3) homeless as defined by lacking “a fixed, regular, and adequate nighttime residence,” and sleeping in a Chicago-based shelter for at least 4 nights of the past week. In the initial phase of the study, we had the additional inclusion criterion that participants must have had a self-reported history of physical or sexual abuse before the age of 17 years. This criterion of childhood abuse was removed a year and a half into the study (corresponding to 24 participants) to allow us to reach more young adults who could benefit from the program. The exclusion criteria included (1) cognitive impairment that would prevent them from understanding or fully engaging in study procedures, (2) involvement in any psychotherapy or legal proceedings that would impact participation, and (3) significant suicidal ideation or behaviors or alcohol or substance dependence in the past 6 months. The substance dependence exclusion criterion was removed a year and a half into the study (corresponding to 28 participants) as long as participants were able to engage in the study procedures.

### Procedures

This pilot trial was approved by the Rush University Medical Center institutional review board. People interested in participating completed an initial intake interview and, if eligible to continue, were offered participation in the pilot trial. We referred to the developed intervention program as the Stepping Stone Project, which was a name collaboratively developed with young adults in focus groups. More information about the other substantive findings of these focus groups are reported elsewhere [23]. The name Stepping Stone highlighted that this intervention was viewed as an introduction into mental health services that could hopefully serve as a bridge to other services once individuals were engaged. All Stepping Stone participants received a mobile phone (Nexus 5, LG Electronics) preloaded with 3 mental health apps developed at the Center for Behavioral Intervention Technologies (Pocket Helper, Purple Chill, Slumber Time described below), a service and data plan, and 1 month of support from a coach in the form of three 30-min phone sessions, as well as opportunities to contact the coach outside of these sessions by phone and text. Coaches were trained therapists experienced in providing treatment in homeless settings. The apps transmitted data to a secure and encrypted University server, and the phone used for text messaging the participants was a University-owned mobile phone that was password protected and encrypted. Participants were provided with training around best practices for maintaining security of their phones including adding a password. Participants completed assessments at baseline and at 1 month. Participants were compensated for completing assessments in the form of the service and data plan. All participants received 1 month of a service and data plan at first. If they completed the endpoint assessment, they received an additional 5 months of service and data for a total of 6 months. Compensation was not tied to engagement in phone sessions or app use.

### Mobile Phones and Phone Apps

Three apps were preinstalled on all mobile phones before distribution to study participants. All participants were asked to use the phones provided by the study and were provided help transferring over their contacts and other phone data if necessary. The service plans were linked to the phones provided to the participants to reduce the likelihood that participants would use multiple phones during the study period. The preinstalled apps included Pocket Helper, which is a daily survey app developed specifically for this study based on focus group input from homeless young adults from the same shelter network [23] and 2 apps (Purple Chill and Slumber Time) from the IntelliCare suite [24,25]. IntelliCare is a collection of mini-apps focused on specific behavior change strategies. We describe these apps in more detail below.

#### Pocket Helper

**Figure 1:**
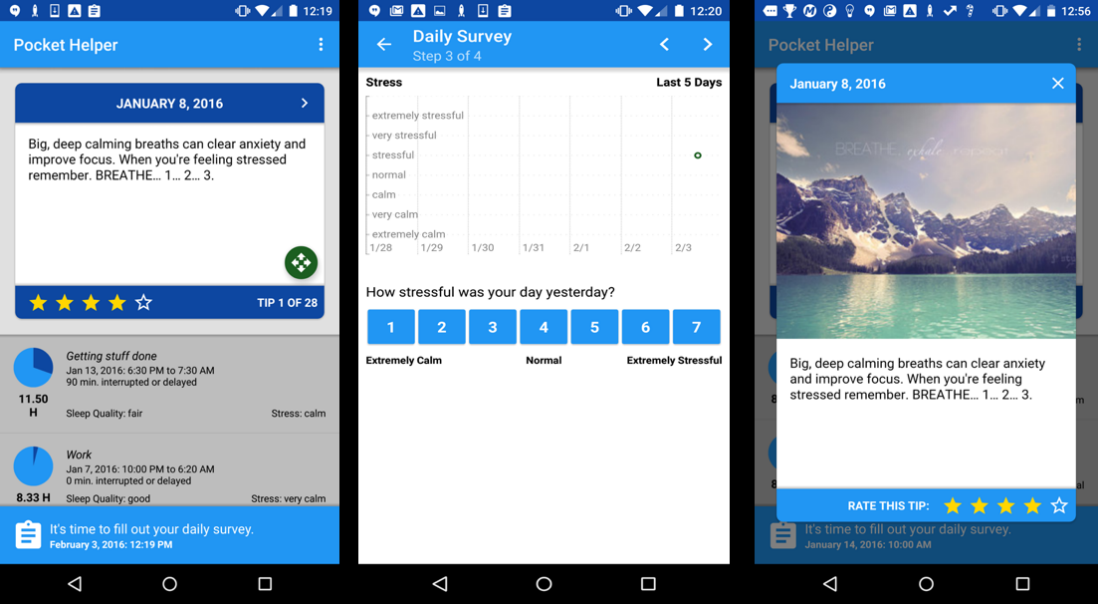
Screenshots of the Pocket Helper app.

Pocket Helper is an app that consists of a daily survey and a daily coping skills–focused tip. Data entered into the app were displayed as feedback to the participant and sent to a coach dashboard. Figure 1 displays screenshots from the app highlighting these main features. The daily survey was intended to take no more than 5 min to complete and asked questions about a participant’s stress level, sleep duration and quality, and the biggest challenge they faced that day. Each daily tip drew randomly from a set of 30 tips that focused on various coping strategies or motivational messages. Tips were sent to participants as push notifications and were also viewable from the home screen when the app was launched. Most tips were associated with either a picture or video that were displayed when the push notification was triggered or could be viewed within the app by expanding the view of the tip from the home screen.

**Figure 2:**
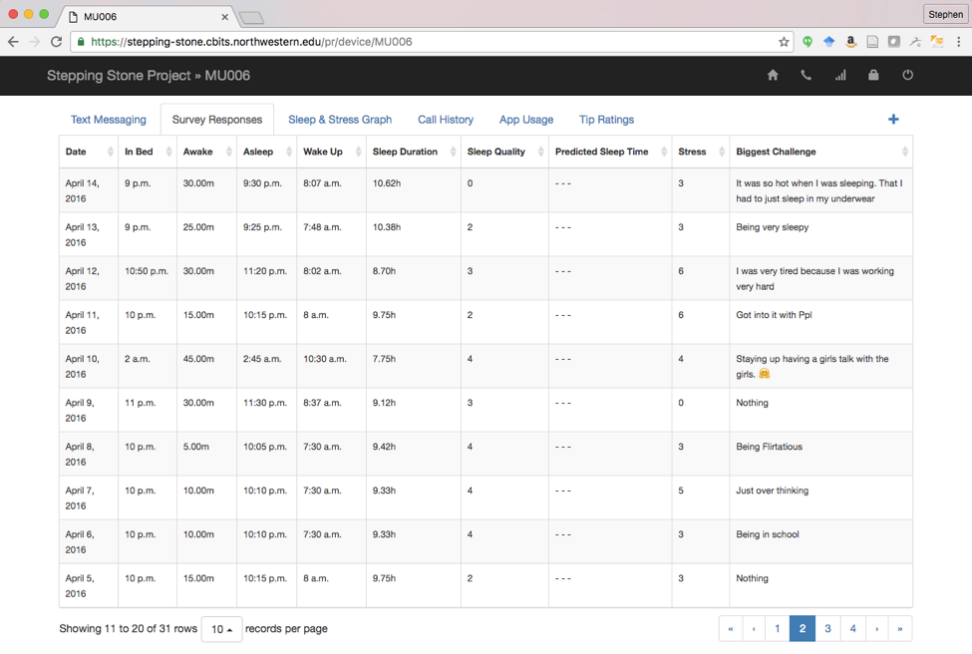
Coach dashboard for the Pocket Helper app.

Pocket Helper was also accompanied by a coach dashboard (Figure 2) that provided the coach with real-time updates regarding information entered into the app. Coaches could view results from a participant’s daily surveys as well as their ratings of coping skill tips. The intention of the dashboard was to allow the coach to incorporate this information into their phone sessions, to use this information as context for the text messages, and to identify the types of skills that might be helpful for each participant based on their needs (stress level and challenges) and interests (responses to daily tips).

#### IntelliCare Apps

IntelliCare is a modular treatment suite consisting of 13 mini-apps, each focused on a singular behavior change technique drawn from CBT and positive psychology [24,25]. IntelliCare has been shown to be effective at reducing depression and anxiety in an 8-week single-armed field trial where participants received the IntelliCare app suite along with brief coaching to support engagement [25]. We selected a subset of the IntelliCare apps that were consistent with content included in the Pocket Helper app as well as that reinforced through the manual for the phone sessions. The 2 IntelliCare apps selected for inclusion in this study were Purple Chill and Slumber Time. Purple Chill provides users with a library of audio recordings that draw from mindfulness, progressive muscle relaxation, deep breathing, and imagery exercises. The goal of Purple Chill is to help promote relaxation and mindfulness practices to reduce stress and worry. Slumber Time prompts the user to complete sleep diaries to track sleep. It provides users with a bedtime checklist based on evidence-based sleep hygiene strategies. It also includes audio recordings to facilitate rest and relaxation and an alarm clock feature to facilitate sleep tracking.

### Phone Sessions and Coaches

All participants were eligible to receive up to three 30-min sessions with the coach over the course of 1 month; these sessions were conducted over the phone. The first session was either scheduled during the onboarding procedure conducted in the shelter, or the coach contacted the participant within 24 hours of onboarding to provide a brief introduction into the schedule and protocol for sessions. Subsequent sessions were scheduled either during the first session or via call, text, or email. These sessions were designed to help provide coping skills to participants in line with CBT principles. We used a modular manualized format in which we outlined the general structure of the 3-session format and identified specific skills and strategies that could be provided based on a participant’s needs and preferences.

The session format was as follows: session 1 consisted of orientation and identification of goals, problems, and resources; session 2 consisted of a check-in on progress and a dedicated focus on a specific topic or skill; and session 3 consisted of reviewing progress and discussing steps for moving forward. The skills and strategies outlined in the manual included the following: psychoeducation, problem solving, mindfulness, relaxation, emotion regulation, imagery rehearsal, sleep hygiene, distress tolerance, interpersonal effectiveness, and safety planning. Session content drew from principles of cognitive behavioral approaches; the session structure was not based on any particular treatment but drew from our experience in designing and delivering mobile-based mental health treatments. Participants were also instructed that they could text message the coach at times outside of these scheduled sessions or set up a brief check-in call of approximately 10 to 15 min. For our initial participants, these consisted of *Virtual Office Hours* in which the coach set aside 1 hour each weekday during which participants could call or text with the coach and receive either 15 min of time over the phone or up to 5 text messages per office hour. After 5 participants, however, we found that few participants were making use of these *Virtual Office Hours*, and we received feedback that this was because the times set each day were not convenient for the participants. As such we moved to a model where participants could text message coaches or set up a brief check-in at times outside of their scheduled sessions and would receive an answer within working hours within no less than 24 hours (except on the weekends).

Sessions, phone, and text messaging support were provided by clinical psychology postdoctoral fellows (AKR and CLD). These fellows underwent weekly supervision where they provided updates on their current participants, the activities in the phone sessions, and any outreach made via text messages. The content of all text messages was transcribed and stored, and coaches completed a form outlining their activities during the phone sessions to indicate the skills and strategies that were covered.

### Assessment and Measurement

Participants completed a series of self-report assessments during enrollment and again at the conclusion of the 1-month intervention.

#### Demographics

Demographic information was collected using a 20-item questionnaire developed by the study team. This was administered only at the baseline session. Variables assessed included age, race and ethnicity, gender, sexual orientation, educational, employment, homelessness status, and history of head injuries.

#### Experience With Technology

Current technology usage habits were assessed using a 10-item Technology Questionnaire. This was administered only at the baseline session. This questionnaire was created by the study team and asked individuals to respond to items indicating the types of devices used (ie, desktop computer, laptop computer, tablet, and mobile phone), and how often they have access to a mobile phone, phone reception, and Wi-Fi. Items also asked how often they used texting, email, and mobile apps, and what incentives and barriers there were to using telemental health resources.

#### Depression

Current depressive symptoms were assessed using the 9-item Patient Health Questionnaire (PHQ-9) [26]. The PHQ-9 asks participants to rate how 9 symptoms have affected them in the past 2 weeks. Response options range from 0 (*Not at all*) to 3 (*Nearly every day*). Higher scores indicate greater symptom severity.

#### Emotion Regulation

Adeptness in identifying and regulating emotions was assessed using the 36-item Difficulties in Emotion Regulation Scale (DERS) [27]. The DERS asked participants to respond to items indicating their level of introspection regarding their emotions and their thoughts and behaviors when they feel upset. Response options range from 1 (*Almost never*) to 5 (*Almost always*). There are 6 subscales including nonacceptance of emotional response, difficulty engaging in goal-directed behavior, impulse control difficulties, lack of emotional awareness, limited access to emotion regulation strategies, and lack of emotional clarity. Eleven items are reversed scored, and higher scores indicate greater problems with emotion regulation.

#### Post-Traumatic Stress Disorder

Current symptoms of PTSD were assessed using the 20-item PTSD Checklist for Diagnostic and Statistical Manual of Mental Disorders-5 (PCL-5) [28]. The PCL-5 is a self-report measure that asks individuals to rate symptoms in the past month based on 1 event that causes them the most distress. Items are rated on a scale of 0 (*Not at all*) to 4 (*Extremely*). Higher scores indicate greater symptom severity.

#### Experience With Mental Health Treatment

Lifetime experience with mental health and psychiatric treatment were assessed using a 19-item Treatment Questionnaire, developed by the study team. Individuals responded to questions asking if they had ever, or if they currently, received one-on-one therapy, attended support groups, used self-help resources (books, websites, and mobile phone apps), or taken medication for psychological problems.

#### Trauma Exposure While Enrolled in Study

Participants were asked to report whether or not they had been exposed to 12 different types of traumatic events during the 1-month study period using a modified version of the Traumatic Events Questionnaire [29]. Specifically, participants were asked about their exposure to the following events *since beginning the study*: a serious transportation accident; serious fire or explosion; serious accident at work, home, or during recreational activity; a natural disaster; physical assault; sexual assault; an unwanted or uncomfortable sexual experience; combat; danger of losing their life or being seriously injured; witnessing someone who was mutilated, seriously injured, or violently killed; or receiving the news of the mutilation, serious injury, or violent or unexpected death of someone close. Item 13 allowed participants to describe any other very traumatic events that occurred during the study period that were not accounted for by the events listed. Participants were considered to have been exposed to trauma during the study period if they said yes to any of the 12 items or said yes to item 13 and provided a sufficient description of a potentially traumatic event.

#### Program Feedback

Feedback on and satisfaction with various aspects of the study (ie, coaching sessions, daily tips, daily surveys, and IntelliCare apps) were assessed using a 20-item Feedback Questionnaire created by the study team. This was administered only at the endpoint session. Satisfaction with the different aspects of the study was indicated on a 5-point Likert scale. Open-text responses were also provided for participants to describe what they liked most and what they liked least about the coaching sessions, office hours (or contact with the coach outside of scheduled sessions), and the Pocket Helper app. Participants were also asked to give suggestions to improve the study.

### Data Analysis

Due to the challenges associated with remote data collection, we instituted several procedures to ensure data quality including attempts to reduce missing data by having research staff follow up with participants, incentivizing completion of questionnaires, and offering to set up in-person meetings to promote survey completion. We also attempted to evaluate the validity of the completed self-report measures. Included in both the baseline and follow-up survey were 4 validity questions. These validity question items were placed at the end of a survey and asked the participant to respond with a certain answer. For example, a validity question embedded in the PCL-5 stated “For this question, please select the answer ‘A little bit’.” If an individual completed more than 1 of these incorrectly, their data were considered to be invalid. For clinical outcomes, which were based on self-report questionnaires, we analyzed data from the 22 participants who completed the study and provided valid responses to both the pre- and postassessment. For satisfaction data, which were completed only at the postassessment, we reported on data from all 26 participants with a valid postsurvey. Because participants received the mobile phones, apps, and phone sessions regardless of providing self-report data, we included all participants’ reports of their use of the apps, sessions, and text messaging to demonstrate feasibility of working in this population.

## Results

### Sample

**Figure 3:**
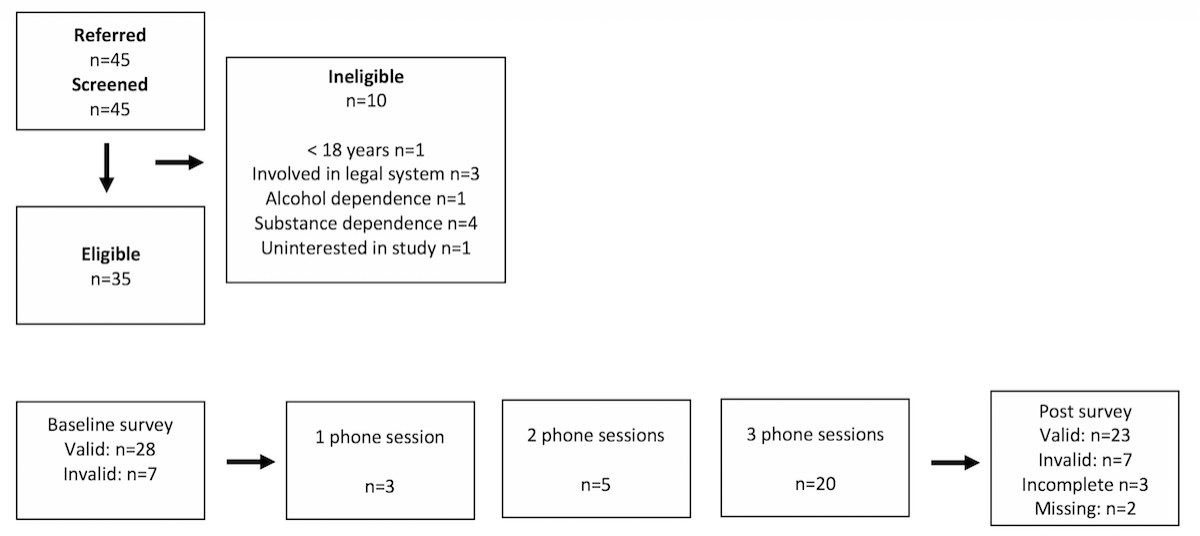
Participant flow through recruitment and intervention.

A total of 35 participants consented and were enrolled in the field trial. Figure 3 outlines the recruitment flow as well as engagement with the intervention. These participants had an average age of 19.06 years (SD 0.85). Most of the participants were women (23/35, 65%), with 31% (11/35) men and 1 transgender (1/35, 3%). Participants were predominantly African American (23/35, 66%), 9% were white (3/35), 17% mixed race (6/35), 3% other (1/35), and 6% either refused or did not know (2/35). In addition, 20% of the sample reported being Hispanic (7/35). The demographics of these homeless youth are reflective of national trends, which show disproportionate ethnic minorities and lesbian, gay, bisexual, transgender, queer, or questioning youth experiencing homelessness [1]. We also asked participants several questions related to educational and occupational attainment and past experiences with homelessness. A total of 21 (21/35, 60%) participants were either in school or employed. Although many of our participants had completed high school (14/35, 40%) quite a few reported less than a high school education (11/35, 31%). Half of the sample reported experiencing homelessness before the age of 18 years (17/34, 50%) with 1 participant noting that their earliest experience with homelessness was at the age of 3 years. Although for many participants this was their first experience with homelessness (13/35, 37%), the rest of participants reported they were homeless between 2 (7/35, 20%) to 10 (2/35, 6%) separate times. We compared those participants who provided valid responses at pre and post as determined by our validity items with those who did not provide valid responses and found no significant differences on any of these characteristics. The clinical characteristics of valid responders at baseline and endpoint are displayed in Table 1.

**Table 1:** Clinical characteristics of sample at baseline and endpoint (1 month).

Outcome	Baseline, mean (SD)	Endpoint, mean (SD)	Probable disorder at baseline, n (%)	Probable disorder at endpoint, n (%)
Depression^a^	11.2 (8.0)	10.1 (8.2)	10 (46)	10 (50)
Post-traumatic stress disorder^b^	32.4 (23.8)	28.2 (23.1)	11 (50)	9 (42)
Emotion regulation	88.9 (30.6)	87.0 (34.6)	No clinical cutoff exists	No clinical cutoff exists

^a^Clinical cutoff for probable depression ≥ 10.

^b^Clinical cutoff for probable post-traumatic stress disorder ≥ 33.

We also assessed participants’ access to and use of various technologies at baseline including computers, mobile devices, and the internet. Most of our participants had mobile phones (25/35, 71%) before receiving one as part of participation in this study, although the type of mobile phone varied considerably. Participants also had access to a variety of other devices including desktops (24/35, 69%), laptops (21/35, 60%), and tablets (19/35, 54%). Similarly, most participants reported having cellular access (20/35, 57%) and access to Wi-Fi for at least half of the waking day (21/35, 60%) although it is worth noting that 20% of our participants (7/35) noted they did not have Wi-Fi access for any part of the day. Use of text messaging and email appeared to be similarly high with 63% (22/35) endorsing texting at least a few times per day and 60% (21/35) endorsing checking email at least a few times per day. Thus, although internet access and use is not ubiquitous, it is quite high.

Few of our participants reported either current or past experience with mental health treatments, with only 17% (6/35) engaged in current and 37% (13/35) engaged in past individual therapy, 3% (1/35) engaged in current and 31% (11/35) engaged in past group therapy, and 6% (2/35) receiving current and 23% (8/35) receiving past medication. Thus, according to self-report, this is a fairly treatment-naïve population.

### Session Attendance and App Use

Most participants (20/35, 57%) completed all 3 of their phone sessions with an average of 2.09 sessions (SD 1.22) completed by each participant. The complete breakdown of the number of sessions completed by participants is provided in Figure 3. It is worth noting that the distribution of sessions was not normally distributed, as the second most frequent number of sessions completed was zero (7/35, 20%). Participants sent an average of 15.06 text messages (SD 12.62) to the coach during the 1-month period or just fewer than 4 text messages per week. Coaches sent slightly more with an average of 19.34 text messages (SD 12.70) or just under 5 text messages per week. There was a correlation between the number of sessions attended and the number of text messages a participant sent (*r*=.35, *P*=.04) but not the number of text messages the coach sent (*r*=.28, *P*=.11). We also examined whether valid responding on the validity items corresponded to increased engagement either in the number of phone sessions attended or the number of text messages sent. Participants who answered the validity items incorrectly attended significantly fewer sessions (mean 1.23) than participants who answered the validity items correctly (mean 2.59, *t*_33_=−3.74, *P*=.001). This was not the case for text messages sent by participants, *t*_33_=−0.62, *P*=.54.

As mentioned previously, we also had our coaches track session content using a reporting form with codes drawing from the topics included in our manual. A total of 73 sessions were provided, and 66 had codes. Of the sessions with codes, 17 had multiple codes with 16 including 2 codes and 1 including 3 codes resulting in 84 total codes across all sessions. The sessions most commonly addressed interpersonal issues (16/84, 19%) or stress management (15/84, 18%). Other common topics included goal setting (9/84, 11%), emotion regulation (8/84, 10%), and family conflict (6/84, 7%). Consistent with our skills-focused approach, very few sessions covered exclusively psychoeducation (1/84, 1%).

### Satisfaction

Satisfaction with the intervention was high, with 100% (23/23) of participants indicating that they would recommend the study to someone else and 52% (12/23) reporting that they were *very* or *extremely* satisfied with the study and the Stepping Stone project. However, less than half indicated they thought it was helpful (10/23, 43%), so it is unclear what their rationale for satisfaction and the recommendation were based on. The most popular component of the intervention was the daily tips, with 64% (14/22) indicating they liked them *quite a bit* or *a lot*. Participants were less enthusiastic about the IntelliCare apps (6/23 or 26% indicating liking them *quite a bit* or *a lot*). The coach support (11/23, 48%), and the office hours (10/23, 43%) were also less popular than the daily tips but received higher satisfaction rating than the IntelliCare apps.

Almost half of participants found the skills they learned in session to be beneficial (11/23, 48%), and almost as many report that they regularly used the skills (10/23, 43%). The intervention length was deemed appropriate by most participants with 56% (12/23) indicating that the 1-month intervention was *just right*. Use of skills learned during coaching sessions was significantly positively related to satisfaction with Pocket Helper (*r*=.78, *P*<.001) and other skills apps (*r*=.46, *P*=.03).

### Feasibility and Safety

We logged all issues that occurred with regard to mobile phones provided to participants. Mobile phone loss (through damage, theft, or other loss) was an anticipated event in this study, and we budgeted mobile phones such that we would be able to replace phones of participants if problems occurred. Overall, we replaced 4 mobile phones during the study period. Two of these phones were replaced because of theft, and 2 phones were replaced because of issues with the device. Issues with the device were a result of our mobile service provider switching carriers, which resulted in complications with access to service. This represents a phone replacement rate of 11% (4/35) and a theft rate of 6% (2/35). The cause of phone loss was obtained through self-report, with the exception of replacements because of issues with mobile service, which was validated independently by our research team through communication with the mobile service provider.

In addition to mobile phone loss, we also closely monitored the use of mobile phone data. All participants received a service plan consisting of unlimited calls and text messages and a data plan consisting of 5 GB of data per month. Although we encountered some issues with participants exceeding this data limit, most participants were able to adopt strategies to allow them to remain within this data cap including monitoring data use on their device and switching to Wi-Fi when secure networks were available. During a period where data caps were temporarily suspended, we did have 1 participant use over 100 GB of data mostly because of streaming videos and music, which demonstrates the tendency to use devices as entertainment devices, which can result in large data demands.

### Clinical Outcomes

Participants experienced very little change on clinical outcomes with small effect sizes for symptoms of depression, *d*=0.27, and effect sizes of *d*=0.17 for symptoms of PTSD and *d*=0.10 for emotion regulation. Given the small sample size, none of these changes were significant (all *P* s>.50). Notably, 45.5% (10/22) of the participants reported exposure to trauma during the 1-month intervention period. Exposures to traumatic events included the following: 18.2% (4/22) serious transportation accident; 18.2% (4/22) physical assault; 13.6% (3/22) serious danger of death or serious injury; 13.6% (3/22) witnessed someone who was mutilated, seriously injured, or violently killed; 9.1% (2/22) serious accident at work, home, or during recreational activity; 9.1% (2/22) received news of the mutilation, serious injury, or violent or unexpected death of someone close; and 4.5% (1/22) natural disaster (note, some individuals had exposure to multiple events). We conducted a post hoc analysis to determine if trauma during the intervention period affected clinical outcomes. Using a multivariate repeated measures analysis of covariance, there were no significant differences in clinical change between those who did and did not have trauma exposure (*F*_3,16_=2.44, *P*=.10); however, there did appear to be a different pattern in changes. Given the small sample size of this study and the resultant statistical power, we will still comment on these changes as exploratory analyses. In this regard, we noted that on average, those who had not experienced a traumatic event during the intervention period had a reduction in PTSD symptoms (mean decrease 6.42, SD 22.01), almost no change in depressive symptoms (mean increase 0.33, SD 3.89), and slightly poorer emotion regulation from pre- to posttreatment (mean increase 1.00, SD 22.41). Those who experienced a traumatic event had an increase in PTSD symptoms (mean increase 3.78, SD 26.67), *t*_19_=0.96, *P*=.35, *d*=0.42, a very small decrease in depressive symptoms (mean decrease 2.25, SD 6.96), *t*_18_=−1.07, *P*=.30, *d*=−0.49, and slight improvements in emotion regulation (mean decrease 3.89, SD 22.48), *t*_19_=−0.49, *P*=.63, *d*=−0.22.

## Discussion

### Principal Findings

This study is the first attempt to deploy and evaluate a mobile phone–based intervention to address the mental health needs of young adults experiencing homelessness. Our findings demonstrated promising results regarding the feasibility and acceptability of such an intervention. Most participants engaged with the program and reported they were satisfied with their participation, although less than half reported that they found the program helpful. Our goal in this study was to establish whether it would be possible to consider digital interventions as a means to bridge individuals until they are ready for care or act as adjunctive elements to more traditional treatment.

Although this pilot feasibility trial was neither designed nor powered to determine the efficacy of the intervention on clinical outcomes, our exploratory results suggest several points for consideration in future studies. For example, most participants experienced only slight improvements in symptoms of depression, PTSD, and emotion regulation as indicated by small effect sizes quantifying the change. We found larger benefits in PTSD symptoms for those who did not experience traumatic events during the course of our 1-month intervention, but those participants also had slight increases in symptoms of depression and emotion dysregulation. Overall, it seems that our intervention was an attractive way to engage young adults experiencing homelessness, but further work might need to consider proximal outcomes that correspond to long-term outcomes of interest and the most effective interventions to deploy within this style of engagement.

In terms of feasibility, we found that engagement with the program tended to be bimodal with most participants completing either 0 or 3 of the offered phone sessions, although nearly 3 times as many participants completed 3 sessions than 0 sessions. This tends to be quite a different pattern from engagement in technology-based interventions more generally where most people who start do not continue with the intervention after an initial use [30]. Moreover, there were no incentives to participate in the intervention, only to complete the study assessments. This seems to suggest that the young adults found something valuable in these calls to continue to engage and that barriers for dropping out were low (as indicated by those who dropped out without completing any sessions). We also found that participants who did not answer our validity questions correctly were also less likely to engage in the phone sessions. As such, it might be possible to determine who is likely to engage long term through early indicators, potentially even as early as patterns of responding to baseline questionnaires. In our other work with technology-based interventions, we have similarly found that the way people use programs can indicate who is likely to persist over time [31].

In terms of acceptability, we found high levels of satisfaction with the program as a whole as well as many of the individual components. Specifically, the daily tips and Pocket Helper app was viewed positively by the young adults. Interestingly, this was the part of the program that was developed specifically for this population, based on our formative work with residents of the same shelter network [23]. Participants also commented positively on the frequency and length of intervention, although some did request additional flexibility with scheduling the coach support components of our program. This desired flexibility prompted us to change the text messaging support from scheduled *office hours* to text messages as needed because participants indicated that the scheduled times were often not convenient for them. We had initially used this scheduled format as we were concerned that participants might overuse the opportunity to text and thus overwhelm our study coach, but that was not the case and the load of around 4 text messages each week was very much manageable.

Findings for satisfaction and acceptability, however, did not correspond to statistically or clinically significant changes on clinical outcomes. Although these findings might be due to our small sample size, it is also worth considering if other factors were at play. For example, perhaps the participants appreciated the attention and support but did not have enough opportunities or structure in their day-to-day lives to implement the skills learned. Another possibility is that the clinical outcomes overlooked other improvements in well-being, such as sense of connection or validation, that might have been worth exploring. Future work might consider more flexibility in the intervention both in terms of content and dosing as well as exploring alternative means of understanding impact. It is worth considering if the dose of the intervention negatively affected the potential to find benefits as three 30-min sessions over a 1-month period is a relatively light intervention, especially in the context of the stressors and challenges faced by homeless young adults. It might be the case that a longer intervention or an intervention of similar length but targeted at times of high need might be more beneficial for homeless young adults. For example, young adults could be monitored through daily surveys as used in our program, and phone support could be initiated when users face significant challenges such as traumatic events or interpersonal stressors or have needs related to work or school placement. Identifying a treatment strategy that will lead to clinical benefits while remaining feasible for youth is an important direction for future research.

### Limitations and Future Work

Our findings are worth contextualizing with regard to the limitations of our design and methods and the formative stage of this work to inform the delivery of technology-based interventions to meet the needs of individuals experiencing homelessness. First, we focused on quantitative data to assess satisfaction and feasibility, and qualitative data could have helped elaborate some of the satisfaction and feasibility issues, particularly the potentially conflicting results on high satisfaction and low helpfulness. Second, although we had fairly broad eligibility criteria, we did allow for some iteration in these criteria during the study. As noted, we removed the criterion related to substance or alcohol dependence but only after 5 potential participants had already been screened out because of those criteria. Of those 5 participants, however, 3 were also receiving concurrent counseling that would also have made them ineligible for this study. Third, it is impossible to disentangle the impact of the clinical intervention (eg, the apps, phone sessions, and text messaging) from receiving a mobile phone with data service. Access to a mobile device with internet connectivity may be an intervention in and of itself. Some research shows that mobile devices may be used by individuals to help cope with stressful situations and when used appropriately can improve psychological and physiological functioning [32]. Games, music, and communication features can all be used to distract or deal with negative emotions, and young adults talked about using mobile devices in this way in our formative work [23]. Finally, although the mobile apps from the IntelliCare suite received the lowest satisfaction ratings of any aspect of the program, it is worth noting that those apps were not deeply integrated into our intervention. We only used 2 of the 13 available IntelliCare apps and did not have specific coach support focused on engagement with those apps as has been tested elsewhere [25]. It would be worth exploring if a full deployment of IntelliCare could be useful for homeless young adults.

Although our satisfaction ratings might be viewed as somewhat mixed given the positive reviews of the program as a whole, the lower ratings of the coach support and office hours, and the lowest ratings of the IntelliCare apps, it is also worth comparing these ratings with satisfaction with other mental health services, especially among young adults experiencing homelessness. Indeed, past work has demonstrated that homeless young adults have low rates of satisfaction with mental health services and that issues of mistrust must be overcome in engaging these young adults successfully in treatment. Our overall rate of 57% of participants completing all of their sessions compares favorably with outpatient psychotherapy generally [33], which is noteworthy given that individuals experiencing homelessness are likely harder to engage and retain than other populations of those with mental health needs. Future work might consider ways to provided *blended* forms of treatment that combine technology and face-to-face treatments or leverage technology to improve the ability of young adults experiencing homelessness to flexibly and successfully engage with treatment. We also note that we developed a multicomponent intervention with support for some but not all elements. Future work might consider this study as a starting point to contribute to the screening phase for Multiphase Optimization Strategy designs, which aim to combine intervention elements into a package for refinement, optimization, and testing [34].

We again note that the impact on clinical symptoms was small and somewhat disappointing in light of the high levels of engagement and positive evaluation of the program we received from the participants. Future work should also assess more proximal outcomes that might mediate subsequent symptom change. Judging from the topics covered in our sessions, outcomes worth assessing would be interpersonal functioning and social support, emotion regulation and coping, and goal setting and problem solving. It must be noted that many of our participants reported using the skills they learned in the sessions and through the apps, but it would be worth evaluating this. Unfortunately, the impact of our intervention on clinical symptoms was small to moderate, with the exception of symptoms of PTSD for individuals who did not experience traumatic events or depression symptoms for those individuals who did experience traumatic events during the intervention period. As noted previously, it is possible that our intervention did not provide a proper dose of treatment. Another possibility is that the intervention itself, but not the delivery platform, may have been a poor fit for our population. Although we used CBT-based coping strategies based on past work that indicated benefits of cognitive behavioral treatments for individuals experiencing homelessness [18,19], we did not engage in a process of specific population–focused tailoring or adaptation. Our coaches did have experience of more generally providing psychotherapy to homeless populations; however, the modularized, brief support provided through our intervention differed from their other clinical experiences. It might be worthwhile to engage in a process of tailoring modularized or common treatment approaches, similar to that we used, to homeless young adult populations. Such approaches have been used in various low-resource settings such as low- and middle-income countries and have demonstrated promising results [35,36].

### Conclusions

This pilot trial of a mobile phone–based mental health intervention for homeless young adults provides mixed support for the promises of such an intervention moving forward. On one hand, we found high rates of engagement and satisfaction, which is noteworthy especially among a population that typically has low rates of trust and satisfaction with mental health services. On the other hand, we found only small benefits in symptoms of mental health issues such as depression, PTSD, and emotion regulation. Given this was a pilot study focused on feasibility and acceptability, we caution against over interpretation of these findings. Nevertheless, we would remiss if we did not note that the extent of changes in clinical symptoms were smaller than we had hoped. We think future work could consider more proximal outcomes to clinical symptom change, as well as other outcomes linked to intervention engagement such as interest in mental health resources, mental health literacy, or mental health stigma. In addition, future research should consider whether intervention content or dose could be modified to be more appropriate for the platform we developed. As such, we believe the major contribution of this study is that it reveals important lessons about engaging homeless young adults using mobile technology. These lessons include the benefits of consistent outreaches (ie, tips and text messaging) as well as the success of providing mobile phones and remote interventions without significant safety issues or loss of devices. We hope further studies continue to explore best ways to create engaging and impactful mental health interventions for homeless young adults and believe this study demonstrated potential ways that technology could play an important role in improving mental health care access for this population.

## Abbreviations

CBT: cognitive behavioral therapy
DERS: Difficulties in Emotion Regulation Scale
PCL-5: PTSD Checklist for Diagnostic and Statistical Manual of Mental Disorders-5
PHQ-9: 9-item Patient Health Questionnaire
PTSD: post-traumatic stress disorder
